# Lightweight tomato ripeness detection algorithm based on the improved RT-DETR

**DOI:** 10.3389/fpls.2024.1415297

**Published:** 2024-07-05

**Authors:** Sen Wang, Huiping Jiang, Jixiang Yang, Xuan Ma, Jiamin Chen, Zhongjie Li, Xingqun Tang

**Affiliations:** ^1^ School of Information Engineering, Minzu University of China, Beijing, China; ^2^ Key Laboratory of Ethnic Language Intelligent Analysis and Security Governance of MOE, Minzu University of China, Beijing, China

**Keywords:** tomato, ripeness recognition, deep learning, RT-DETR, PConv, deformable attention, slimneck, Inner-EIoU

## Abstract

Tomatoes, widely cherished for their high nutritional value, necessitate precise ripeness identification and selective harvesting of mature fruits to significantly enhance the efficiency and economic benefits of tomato harvesting management. Previous studies on intelligent harvesting often focused solely on identifying tomatoes as the target, lacking fine-grained detection of tomato ripeness. This deficiency leads to the inadvertent harvesting of immature and rotten fruits, resulting in economic losses. Moreover, in natural settings, uneven illumination, occlusion by leaves, and fruit overlap hinder the precise assessment of tomato ripeness by robotic systems. Simultaneously, the demand for high accuracy and rapid response in tomato ripeness detection is compounded by the need for making the model lightweight to mitigate hardware costs. This study proposes a lightweight model named PDSI-RTDETR to address these challenges. Initially, the PConv_Block module, integrating partial convolution with residual blocks, replaces the Basic_Block structure in the legacy backbone to alleviate computing load and enhance feature extraction efficiency. Subsequently, a deformable attention module is amalgamated with intra-scale feature interaction structure, bolstering the capability to extract detailed features for fine-grained classification. Additionally, the proposed slimneck-SSFF feature fusion structure, merging the Scale Sequence Feature Fusion framework with a slim-neck design utilizing GSConv and VoVGSCSP modules, aims to reduce volume of computation and inference latency. Lastly, by amalgamating Inner-IoU with EIoU to formulate Inner-EIoU, replacing the original GIoU to expedite convergence while utilizing auxiliary frames enhances small object detection capabilities. Comprehensive assessments validate that the PDSI-RTDETR model achieves an average precision mAP50 of 86.8%, marking a 3.9% enhancement over the original RT-DETR model, and a 38.7% increase in FPS. Furthermore, the GFLOPs of PDSI-RTDETR have been diminished by 17.6%. Surpassing the baseline RT-DETR and other prevalent methods regarding precision and speed, it unveils its considerable potential for detecting tomato ripeness. When applied to intelligent harvesting robots in the future, this approach can improve the quality of tomato harvesting by reducing the collection of immature and spoiled fruits.

## 1 Introduction

Tomatoes are widely favored for their rich content of vitamin C, potassium, and lycopene (Story et al., 2010). During the growth of tomatoes, their color gradually shifts from green to yellow, orange, and finally red, while the firmness decreases, and the sweetness and acidity reach a balance, leading to an increase in nutritional content. The maturity level of tomatoes directly impacts their nutritional value, taste, and the timing of harvesting. Traditional manual detection methods are subjective and are often inefficient and costly, failing to meet the needs for high-efficiency maturity discrimination and harvesting (El-Bendary et al., 2015). Although sensors provide a non-contact means of detecting maturity, they sometimes struggle to accurately distinguish between closely related stages of ripeness, are significantly influenced by ecological elements like illumination and temperature, and are cost-prohibitive (Aghilinategh et al., 2020; Alam Siddiquee et al., 2020). Consequently, the creation of a lightweight, efficient, and precise algorithm for maturity detection is of great importance for the intelligent grading and harvesting of tomatoes However, the natural growth environment of tomatoes, characterized by fruit occlusion, subtle color differentiation, and variations in lighting conditions, presents challenges for the accurate identification of tomato maturity.

To enhance the quality of tomato harvests and reduce labor costs, while also accurately distinguishing fruits of different maturities for harvesting, certain conventional machine learning methods have been utilized in the maturity detection of fruits and vegetables. For example (Zhao et al., 2016), extracted Haar-like characteristics from monochrome images of tomatoes and used an AdaBoost classifier to identify potential tomato targets, but the precision and speed of their identification still require enhancement (Liu et al., 2019). utilized the Gradients of Oriented Histograms descriptor for training the classifier and introduced a coarse-to-fine scanning technique that enhanced the accuracy of tomato detection; however, the algorithm did not consider maturity grading and was limited only to the identification of ripe tomatoes, which constitutes a significant limitation. Recently (Bai et al., 2023), utilized machine learning to aid in image analysis, merging form, surface, and hue characteristics of tomatoes to achieve high-precision identification and picking point localization of clustered tomatoes. However, their model is large and requires high-end hardware. Although traditional machine learning demonstrates significant advantages over manual inspection in identifying the ripeness of tomatoes, it still suffers from issues such as the cumbersome process of manual feature extraction, high model complexity, low detection accuracy, and slow processing speeds.

In unstructured environments, the characterization of tomatoes is further complicated by factors such as variable lighting conditions, occlusion by leaves, and overlapping fruits. These challenges render conventional machine vision algorithms less effective in differentiating the maturity levels, leading to considerable limitations in their applicability. Consequently, deep learning has been employed to address these aforementioned difficulties (Afonso et al., 2020). utilized the Mask-RCNN (He et al., 2017) algorithm to identify tomato images captured in a greenhouse environment, but its ability to distinguish the ripeness of tomatoes was subpar (Ko et al., 2021). have implemented multi-stream convolutional network (Simonyan and Zisserman, 2014) for the detection of tomato ripeness and have employed probabilistic decision integration to achieve more accurate categorization outcomes of ripeness, thereby achieving commendable detection accuracy. Nonetheless, the research pertaining to the real-time identification of tomatoes remains deficient. In recent years, models from the YOLO (Redmon et al., 2016; Redmon and Farhadi, 2017) series have demonstrated exceptional effectiveness within the realm of industrial object recognition, outperforming conventional two-stage detection pipelines (Liu et al., 2020). have substituted conventional rectangular boxes for circular ones for tomato localization, introducing a modified YOLOv3-based detection model that reduces the impact of variations in lighting, overlaps, and obstructions. Nevertheless, this model does not consider ripeness information, which limits its effectiveness in detecting tomatoes across various growth stages (Lawal, 2021). have employed compact architecture integration and pyramid pooling within a modified YOLOv3 framework to enhance the identification accuracy of tomatoes (Zheng et al., 2022). have integrated the ResNet (He et al., 2016) architecture into the CSPDarknet53 backbone of YOLOv4, incorporating depthwise separable convolution blocks as residual edges to execute tomato object detection tasks at three different scales. This has enhanced the robustness under varying degrees of occlusion and lighting conditions. Additionally (Khan et al., 2023), have proposed a novel approach to tomato ripeness classification utilizing a modular convolutional transformer. This method merges the benefits of convolutional networks and transformers (Vaswani et al., 2017), potentially elevating the productivity and correctness in the processes of tomato collection, evaluation, and quality oversight. However, its detection capabilities are limited when dealing with tomatoes that are heavily occluded, cluttered, or small in size.

Deep learning supersedes traditional machine learning in automated feature extraction and handling of high-dimensional data, particularly achieving higher accuracy in image recognition tasks. In the agricultural sector, especially in fruit and vegetable harvesting, there is a growing demand for low-power embedded devices to reduce costs and improve efficiency. Hence, with algorithmic practicality in mind, it is crucial to minimize the footprint and computational demands of the model while concurrently improving the accuracy and velocity of identification. Furthermore, the algorithms are required to possess robustness to withstand interference from unstructured external elements such as changing illumination, climatic conditions, and obstructions due to vegetation. These challenges necessitate the development of innovative deep learning approaches that can effectively balance performance, efficiency, and adaptability to real-world scenarios. This study introduces a lightweight tomato maturity identification approach utilizing an enhanced RT-DETR framework to tackle the previously mentioned possible issues. Our main contributions are as follows:

1. “Integration of residual blocks with partial convolutional”: By amalgamating PConv lightweight convolutions with residual blocks into a novel PConv_Block module, enhanced residual architecture for backbone networks. This integration preserves performance while reducing computational load, thereby elevating the efficiency of feature extraction.
2. “Introduction of deformable attention mechanism”: Incorporating the deformable attention mechanism into the encoder of the transformer to replace the multi-head attention mechanism results in the AIFI-DAT component. This enables the framework to grasp intricate associations among various segments of the input, providing enhanced performance in the task of fine-grained classification of tomato ripeness.
3. “Design of a lightweight Neck architecture”: The novel slimneck-SSFF structure is proposed by integrating the Scale Sequence Feature Fusion (SSFF) framework with the slim-neck, which incorporates lightweight GSConv and VoVGSCSP modules. Introduced at the Neck stage, this architecture boosts the detection abilities for tiny items while preserving precision, and concurrently reduces computational demand and inference latency.
4. “Loss function optimization”: The Inner-IoU is amalgamated with EIoU, introducing an auxiliary bounding box within EIoU controlled by a scale factor ratio to obtain Inner-EIoU. Employing this loss function in place of the original GIoU used by the model yields faster and more efficient regression results.
5. “Evaluation of effectiveness”: Thorough assessment with the tomato maturity dataset shows that the proposed PDSI-RTDETR framework outperforms the initial RT-DETR framework regarding accuracy and speed, with reduced computational costs, and outperforms other common object detection models.

## 2 Materials and methods

### 2.1 RT-DETR network

RT-DETR is a novel real-time end-to-end target detection model (Lv et al., 2023). Compared to YOLOv8, RT-DETR demonstrates improved efficacy and better equilibrium in the same testing environments, with a shortened training period and without employing the mosaic data augmentation strategy, while maintaining detection speeds on par with the YOLO series. The RT-DETR model is partitioned into three primary segments: the backbone network, the hybrid encoder, and the decoder.

**Figure 1:**
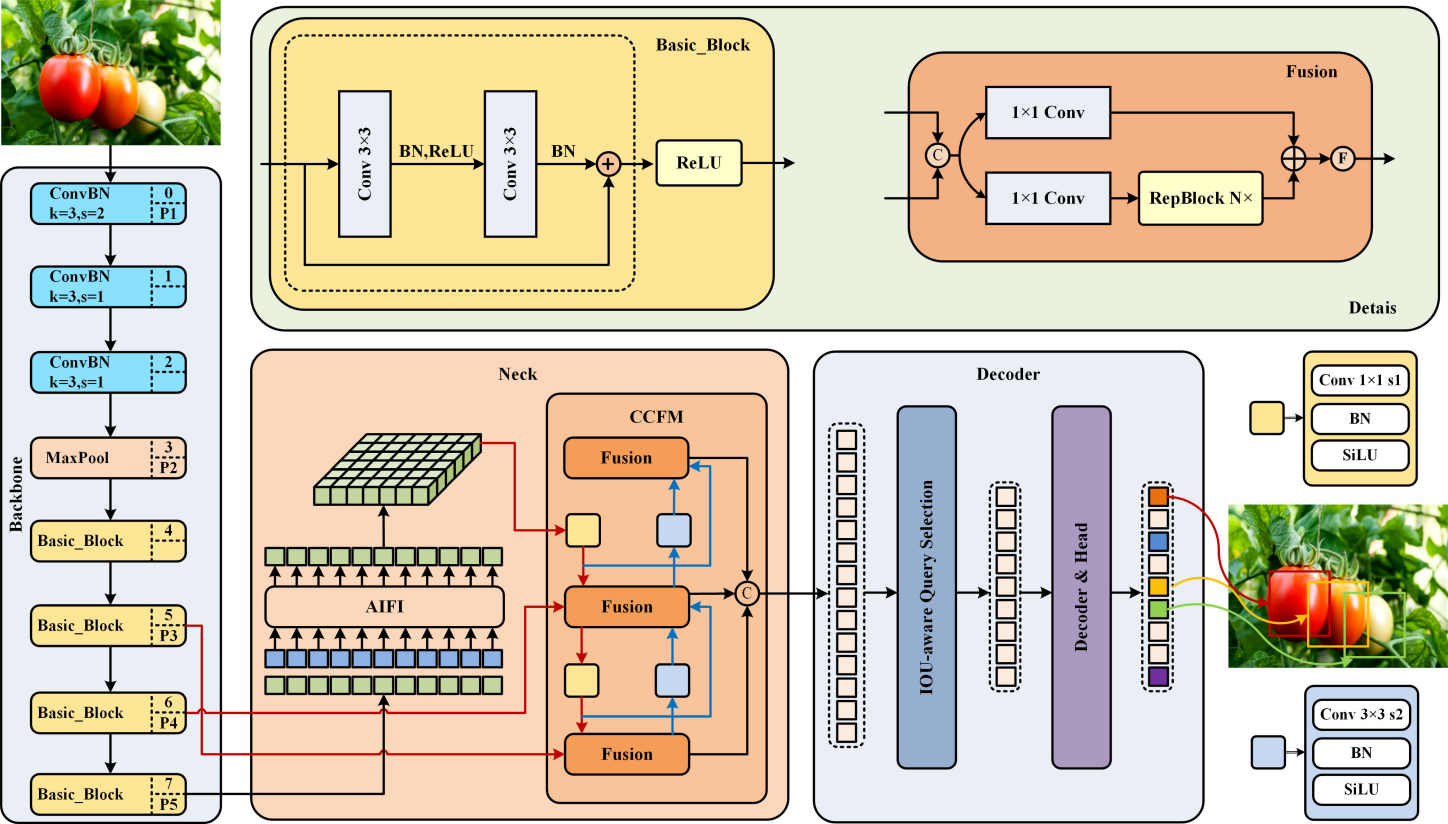
RT-DETR network structure diagram.

The backbone architecture harnesses the capabilities of a convolutional network to extract salient features, procuring outputs across three distinct scales with strides of 8, 16, and 32. The neck network employs an Attention-based Intra-scale Feature Interaction (AIFI) module to process high-level features from the backbone network, significantly reducing computational load and improving computational speed without compromising performance. It also uses a Cross-scale Feature-Fusion Module (CCFM) for the integration and interaction of multi-scale features. RT-DETR leverages a transformer-based denoising decoder to enhance the quality of bipartite matching samples, accelerating the convergence rate during training. The model dynamically adjusts queries based on IoU, focusing efforts on regions most relevant to the detection targets. The decoder obtains initial object queries from an IoU-aware selection mechanism and iteratively refines them. Overall, RT-DETR presents significant potential for advancement in industrial object detection. The structure of the foundational model RT-DETR selected for this article is shown in **Figure 1**.

### 2.2 Dataset collection

**Figure 2:**
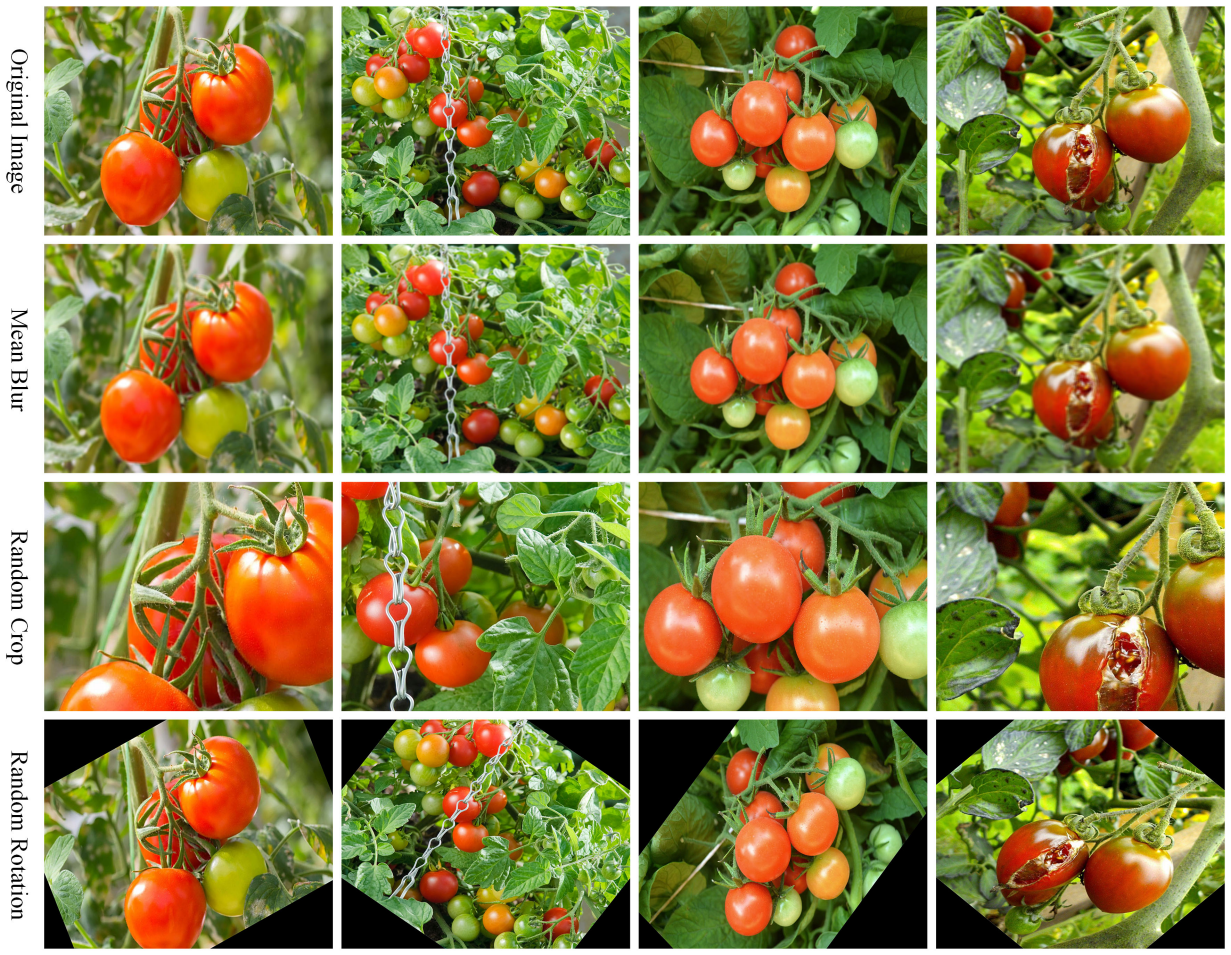
Examples of data augmentation techniques applied to the tomato images.

The main objective of this research is to ascertain the maturity level of tomatoes cultivated in outdoor settings, thus improving the robustness of our model. To enhance data diversity, images of tomato ripeness were acquired in two batches. The first batch of photographs was taken during the daytime in a tomato picking garden located in Fengtai District, Beijing, China (longitude 116°12’3.7548”E, latitude 39°47’26.8332”N), using a Xiaomi 9 smartphone equipped with a SONY IMX586 lens (48 MP, f/1.7 aperture, 26mm equivalent focal length). The images were captured under various lighting conditions, ranging from bright sunlight to overcast skies, with temperatures between 25°C and 30°C in early September. The captured images exhibit varying conditions, such as strong light, shadow occlusion, and overlapping fruits. The second batch of images was sourced from 112 tomato images in the publicly available Fruits and Vegetables Image Recognition Dataset (Seth, 2020) on Kaggle. These images underwent data augmentation processing, including mean blur (with kernel sizes ranging from 5×5 to 15×15), random cropping (cropping a random portion of the image within 40-80% of the original image size), and random rotation (rotation by a random angle between -60° and 60°), to enhance the diversity of the dataset. All augmented images were resized to a consistent size of 640x640 pixels. After data augmentation, a total of 3,251 images were obtained. Examples of data augmentation are shown in **Figure 2**.

To meet the experimental requirements, the dataset was divided into three parts using random splitting. First, the dataset was randomly shuffled to ensure the randomness of the samples. Then, according to the predefined ratios, the shuffled dataset was divided into three subsets: 2,275 images (70%) were used for the training dataset, 325 images (10%) for the validation dataset, and 651 images (20%) were designated for the testing dataset. This random splitting approach effectively assigns the data to different subsets, reducing the bias introduced by the division of the dataset and improving the generalization ability of the model.

### 2.3 Dataset annotation and processing

Given that the principal emphasis of this paper lies in the analysis of computer vision algorithms in assessing tomato ripeness, visual differentiation is performed based on the appearance differences in coloration, hue saturation, size and shape, as well as the degree of spoilage. Based on these characteristics, tomatoes are categorized into five classes: unripe, half-ripe, ripe, overripe, and rotten. Unripe tomatoes exhibit a bright green hue, smaller size, low sugar content, acidic taste, and generally have a total soluble solids TSS content below 5%. Half-ripe tomatoes transition to yellow or pink while retaining green areas, with a sugar content of around 8%, a pH level of approximately 4.25, and a slightly increased TSS of about 7%. Ripe tomatoes are uniformly red or deep red, larger in volume, and vivid in color, with a pH typically ranging between 4.2 and 4.5, and a higher TSS content, likely between 10-12%. Overripe tomatoes are deep red, beginning to lose their luster, showing slight shrinkage or skin relaxation. Rotten tomatoes may develop irregular brown or black spots, lose their normal skin sheen, exhibit significant shrinkage, soften, or even burst. Utilizing the Labelme annotation tool, the tomatoes in the images are manually labeled according to the aforementioned characteristics. Detailed information about the dataset annotation is presented in **Table 1** as follows:

**Table 1:** Tomato ripeness dataset labeling information.

Types	Number	half_ripe	over_ripe	ripe	rotten	unripe
Training	2275	721	1285	2417	980	1408
Validation	325	146	237	337	101	222
Test	651	216	328	627	265	489
Total	3251	1083	1850	3381	1346	2119

### 2.4 Training and experimental comparison platform

The network experimental environment is based on Ubuntu 20.04, Python 3.10.12, and Pytorch 2.0.1, with relevant hardware configurations and model parameters detailed in **Supplementary Table 1**. The batch size was set to 4, with a training duration of 100 epochs, and a learning rate selected at 0.0001. An adaptive image size of 640×640 was selected for the experiments.

## 3 Algorithm design

### 3.1 The PDSI-RTDETR model architecture

**Figure 3:**
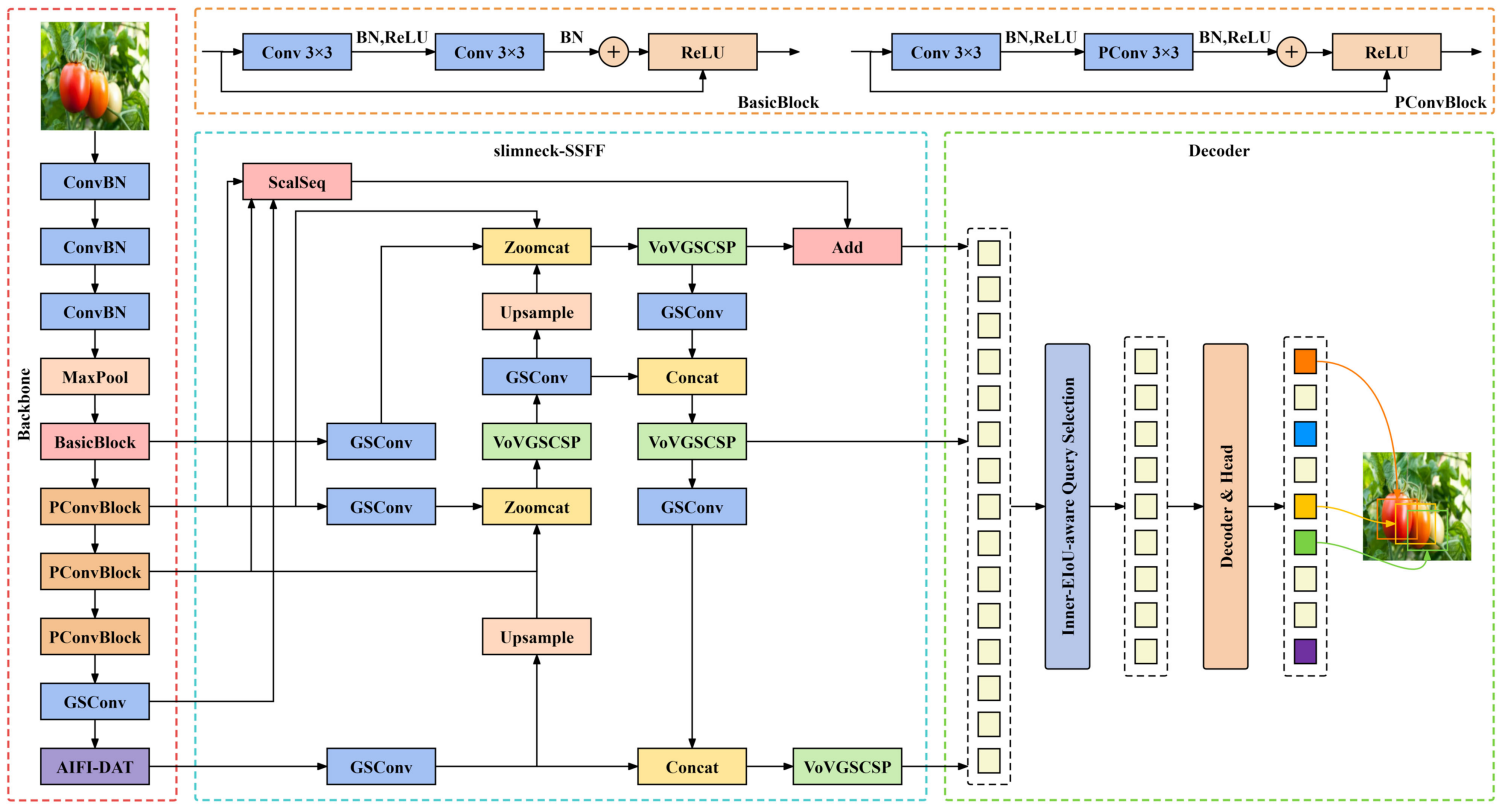
The structure diagram of improved RT-DETR(PDSI-RTDETR).

The advent of RT-DETR has filled a void in the DETR (Carion et al., 2020) series for real-time monitoring applications, offering a superior balance of precision and velocity relative to the YOLO series. On the other hand, the detection of tomato ripeness in natural environments necessitates not only high accuracy and rapid processing but also confronts the challenge of model lightweighting to mitigate issues such as hardware costs. This study introduces the lightweight PDSI-RTDETR model to address these concerns. The PConv_Block module, proposed in this paper, merges partial convolutional (PConv) (Chen et al., 2023) with residual blocks, optimizing the backbone network for efficient feature extraction with reduced computational burden. Deformable attention mechanisms (Xia et al., 2022) are introduced to the encoder, enhancing fine-grained classification through the AIFI-DAT module. The proposed slimneck-SSFF structure combines the Scale Sequence Feature Fusion framework (Kang et al., 2023) with a slim-neck design, featuring GSConv and VoVGSCSP modules (Li et al., 2022a), to improve small object detection with reduced computational cost and lower inference latency. Optimization of the loss function is achieved by integrating Inner-IoU (Zhang et al., 2023a) with EIoU (Zhang et al., 2022) to form Inner-EIoU, improving regression efficiency. Comprehensive evaluations confirm that the PDSI-RTDETR model outperforms the baseline RT-DETR and other prevalent object detection methods in accuracy, speed, and computational efficiency. **Figure 3** illustrates the PDSI-RTDETR model architecture.

### 3.2 Backbone network improvement

To circumvent the computational redundancies of complex models in simple tasks, which result in diminished detection velocities, this paper utilizes the comparatively light ResNet-18 (He et al., 2016) as the baseline for the backbone network. Moreover, we substitute the conventional convolutions within the Basic_Block modules with partial convolutions to bolster feature extraction while simultaneously achieving heightened model lightness. Partial convolution judiciously utilizes filters across a chosen subset of input channels, thereby conserving the remainder, which culminates in lower Floating Point Operations Per Second (FLOPs) than standard convolution. This method secures an elevated operational speed on an extensive spectrum of devices without detracting from the precision of the task. The structural principle of PConv is illustrated in **Supplementary Figure 1**.

The PConv employs conventional convolution on a specific portion of the input channels to derive spatial features, preserving the rest of the channels unchanged. To ensure efficient memory access, the calculation engages the first or last sequence of $c_{p}$ consecutive channels as a proxy for the computational demand across all feature maps. Maintaining methodological uniformity, it’s assumed that the count of channels remains the same for both input and output feature maps. Therefore, the FLOPs of a PConv are only, as detailed in Equation (1):


(1) $$h\times w\times k^{2}\times c_{p}^{2}$$

Within the formula, h and w denote the dimensions of the feature map, k indicates the size of the convolution kernel, and $c_{p}$ is the count of channels utilized by the standard convolution. Typically, with $r=c_{p}/c=1/4$ , the FLOPs for PConv are just 1/16 of a regular Conv. Additionally, the scenario of diminished memory access for PConv includes, as specified in Equation (2):


(2) $$h\times w\times 2c_{p}+k^{2}\times c_{p}^{2}\approx h\times w\times 2c_{p}$$

**Figure 4:**
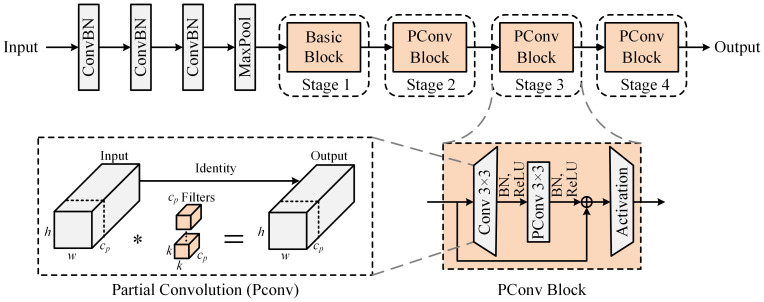
Lightweight feature extraction backbone network structure incorporating PConv.

Memory access for PConv is merely a quarter compared to a typical convolution, as the remaining $c∼c_{p}$ channels do not participate in the computation and therefore do not require memory access. Incorporating PConv into the feature extraction network markedly decreases computational and memory demands, streamlining the backbone network and enhancing model inference speed. The backbone network incorporating the proposed PConv_Block module is shown in **Figure 4**.

### 3.3 Improvements to the efficient hybrid encoder

#### 3.3.1 Deformable attention

**Figure 5:**
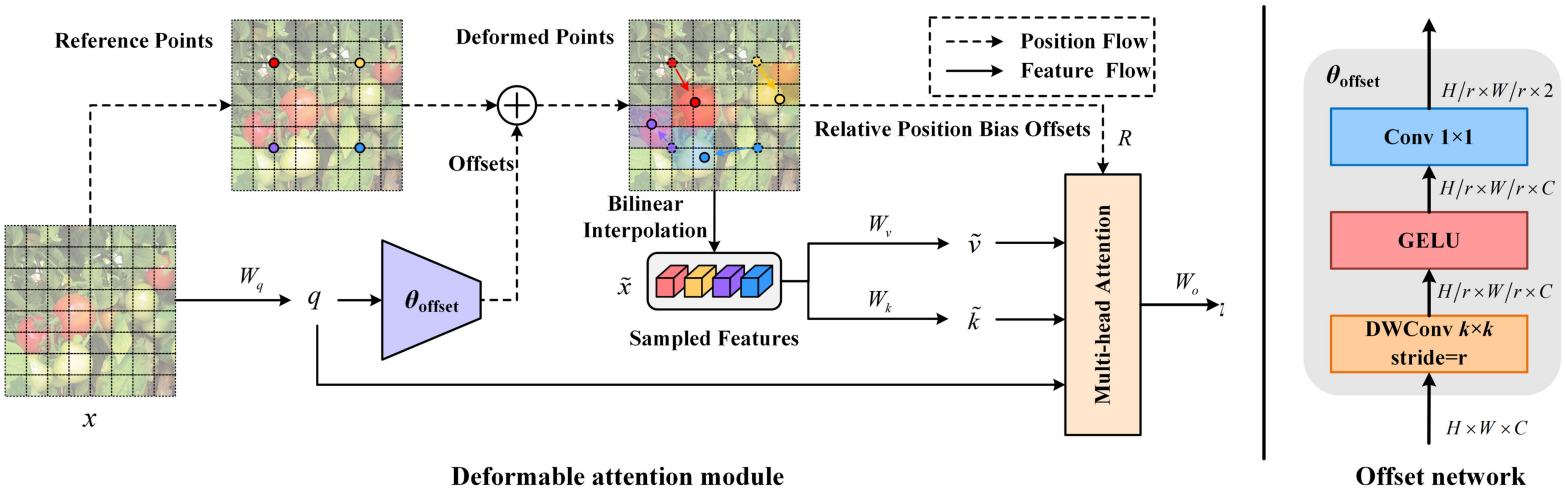
Deformable attention mechanism structure, which adjusts reference points and applies relative position bias through an offset network to optimize feature transformation.

The deformable self-attention module determines the locations of key and value pairs inside the self-attention framework based on the data. This method facilitates targeted attention on significant areas, enhancing the extraction of meaningful features. Additionally, it addresses the issue of excessive memory and computational costs associated with dense attention. Deformable attention shares shifted keys and values for each query, reducing spatial complexity and avoiding significant information loss that can result from down-sampling techniques. An illustration of deformable attention is presented in **Figure 5**.

As shown above, for an input feature map $x\in {\mathbb{R}}^{H\times W\times C}$ , a uniform grid of points $p\in {\mathbb{R}}^{H_{G}\times W_{G}\times 2}$ is established as a baseline. To calculate the offsets for each grid point, the feature map undergoes a linear transformation into query tokens $q=xW_{q}$ , which are then processed by a specialized lightweight network $\theta_{offset}$ to produce the offsets $\text{Δ}p=\theta_{offset}\left(q\right)$ . Following this, sampling of features occurs at these adjusted points to form keys and values, culminating in the creation of the projection matrix, as described in Equations (3) and (4):


(3) $$q=xW_{q},\tilde{k}=\tilde{x}W_{k},\tilde{v}=\tilde{x}W_{v}$$

(4) $$\text{Δ}p=\theta_{offset}\left(q\right),\tilde{x}=\emptyset \left(x;p+\text{Δ}p\right)$$

$\tilde{k}$ and $\tilde{v}$ respectively represent the deformed key and value embeddings, and are transformed into bilinear interpolation using the sampling function ∅(·;·) , as defined in Equation (5):


(5) $$\emptyset \left(\text{z};\left(p_{x},p_{y}\right)\right)=\sum_{\left(r_{x},r_{y}\right)}ɡ\left(p_{x},r_{x}\right)ɡ\left(p_{y},r_{y}\right)z\left[r_{y},r_{x},:\right]$$

where ɡ(a,b) and the coordinates $\left(r_{x},r_{y}\right)$ span every position on $z\in {\mathbb{R}}^{H\times W\times C}$ . Multi-head attention is performed on q,k,v , incorporating relative positional displacements R . The result from the attention heads is articulated as follows in Equation (6):


(6) $$Z^{\left(m\right)}=\text{σ}\left(q^{\left(m\right)}{\tilde{k}}^{\left(m\right)T}/\sqrt{d}+\emptyset \left(\hat{B};R\right)\right){\tilde{v}}^{\left(m\right)}$$

where σ(·) represents the softmax operation and d is the dimensionality of each attention head. $Z^{\left(m\right)}$ signifies the output embedding from the m -th attention head. The outputs from all heads are merged via concatenation and then transformed through $W_{o}$ to produce the final output z , as described in Equation (7):


(7) $$Z=Concat\left(z^{\left(1\right)},\cdots ,z^{\left(M\right)}\right)W_{o}$$

#### 3.3.2 Introduce deformable attention into the AIFI module

Within the neck architecture of the model, a single transformer encoder layer is dedicated to processing the $S_{5}$ features emanating from the backbone network. Leveraging the rich semantic attributes of high-level features, this methodology significantly curtails computational demands and augments processing speed without sacrificing performance robustness. This optimized hybrid encoder orchestrates intra-scale feature interaction, morphing multi-scale features into a serialized array of image feature sequences. The replacement of the conventional multi-head self-attention with deformable attention facilitates adaptive sampling of pivotal feature locations, diminishing memory usage and circumventing the severe information loss inherent to down-sampling techniques, thereby elevating the computational efficiency and prowess in feature capture of the model. The computational process, as delineated in Equations (8) and (9), is as follows:


(8) $$Q=K=V=FlDatten\left(S_{5}\right)$$

(9) $$F_{5}=Reshape\left(DAttn\left(Q,K,V\right)\right)$$

where DAttn represents the deformable attention, and Reshape represents the restoration of the feature’s shape to match that of $S_{5}$ , which is the inverse operation of FlDatten .

### 3.4 Improved neck

The role of the neck network in the model is to coordinate and bolster the feature representation at different levels in order to enhance the precision in identifying targets across various sizes. The neck network of the RT-DETR model uses the AIFI module to process the high-level features, and then the CCFM module is used for the interaction and fusion of multi-scale features. Its compared with YOLO the number of parameters and computation of this neck structure of the network has risen, and considering the necessity to detect a significant volume of targets within an individual image and the large color difference between different ripeness of tomatoes, the original model loses the small target information in the process of convolution and down-sampling. Therefore, this paper introduces the SSFF module, GSConv and Slim-neck technology, and proposes the slimneck-SSFF feature fusion architecture, which reduces the complexity and computation of the model on the basis of improving the accuracy.

**Figure 6:**
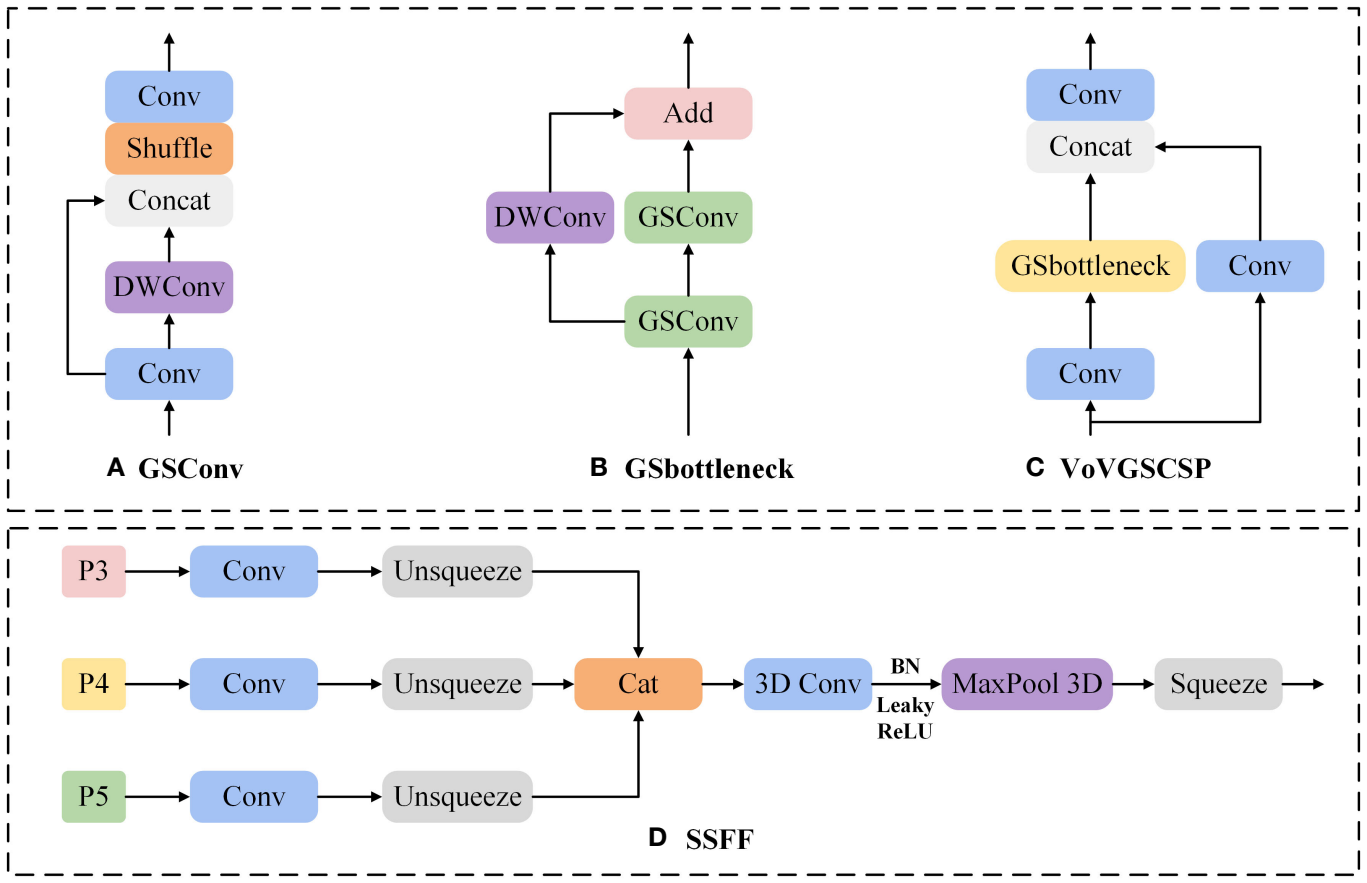
**(A)** GSConv, **(B)** GSbottleneck, **(C)** VoVGSCSPC, and **(D)** SSFF module structure diagrams.

As shown in **Figure 6**, the GSConv module combines conventional convolution with separable convolution and uses the Shuffle procedure to integrate the features generated by both, ensuring inter-channel information exchange while effectively reducing computational costs.

As illustrated in **Figures 6B, C**, GSbottleneck consists of two GSConv modules and one DWConv module, with input features fed into each module and their outputs summed. Based on GSbottleneck, VoVGSCSP is constructed using a one-off aggregation approach, effectively decreasing the count of parameters and floating-point computations.

To distinguish between the ripeness stages of tomatoes across different sizes, we employ the SSFF module to boost the network proficiency in capturing scale-diverse features. The structure of the SSFF module is depicted in **Figure 6D**. The SSFF module treats feature maps of different sizes as a scale space, adjusts the effective feature maps of different resolutions to the same resolution for concatenation, then horizontally stacks the feature maps of different scales, and utilizes three-dimensional convolution to extract their scale sequence features. In contrast to existing literature that merely adopts summation or concatenation methods to fuse pyramid features, the SSFF module can better integrate the high-dimensional information from deep feature maps with the detailed information from shallow feature maps. This provides more comprehensive and refined feature descriptions for objects of different dimensions, thereby enhancing the network’s ability to capture multi-scale features.

### 3.5 The improved loss function

In the research conducted, the original GIoU of the model is substituted with Inner-EIoU, which offers quicker convergence, enhanced accuracy in assessment, and supplementary edges. The use of smaller auxiliary borders to compute the loss during the model training process has a gain effect on the regression of the high IoU samples, and the opposite is true for the low IoU samples. Employing the scale factor ratio to manage the creation of various scales of auxiliary edges for loss calculation yields quicker regression outcomes across diverse scenarios. Applying Inner-IOU to the EIOU loss function is calculated as follows, according to Equations (10)–(14):


(10) $$inter=\left(\min\left(b_{r}^{ɡt},b_{r}\right)-\max\left(b_{l}^{ɡt},b_{l}\right)\right)*\left(\min\left(b_{b}^{ɡt},b_{b}\right)-\max\left(b_{t}^{ɡt},b_{t}\right)\right)$$

(11) $$union=\left(w^{ɡt}*h^{ɡt}\right)*{\left(ratio\right)}^{2}+\left(w*h\right)*{\left(ratio\right)}^{2}-inter$$

(12) $$IoU^{inner}=\frac{inter}{union}$$

(13) $${ℒ}_{EIoU}={ℒ}_{Iou}+{ℒ}_{dis}+{ℒ}_{asp}$$

(14) $${ℒ}_{Inner-EIoU}={ℒ}_{EIoU}+IoU-IoU^{inner}$$

where the ground truth (GT) box and anchor are respectively represented as $B^{ɡt}$ and B . The width and height of the GT box are denoted by $w^{ɡt}$ and $h^{ɡt}$ , while the width and height of the anchor box are represented by w and h . The ratio is an auxiliary factor that controls the size of the helper box. The EIOU loss function is composed of three components: the overlap loss ${ℒ}_{Iou}$ , the center distance loss ${ℒ}_{dis}$ , and the width and height loss ${ℒ}_{asp}$ . Inner-EIoU, focusing more accurately on the box’s center, is an enhanced bounding box regression loss that quickens convergence through loss calculation with scale-modified support boxes and shows efficacy in detecting smaller objects.

## 4 Evaluation indicators

The paper assesses the algorithmic performance by comparing the disparities in image detection by the network model before and after enhancements, under identical experimental settings. The study employs precision (P), recall (R), mean average precision (mAP), F1 score, GFLOPs, and frames per second (FPS) as evaluative criteria.

Precision represents the proportion of true positives in the predictions classified as positive by the model. It gauges the adeptness of the model in distinguishing negative samples. Higher precision denotes greater reliability of the model in predicting positive cases. The formula for precision is presented as in Equation (15).


(15) $$Precision=\frac{TP}{TP+FP}$$

Recall quantifies the fraction of true positive samples accurately detected as positive by the model, showcasing the proficiency in identifying positive instances. An increased recall suggests the model identifies more true positives. The formula for calculating recall is presented in Equation (16).


(16) $$Recall=\frac{TP}{TP+FN}$$

The F1 score, serving as the harmonic mean between precision and recall, aims to consolidate these metrics into one indicator. This score varies between 0 and 1, where scores nearing 1 denote a stronger model. The calculation for the F1 score is detailed in Equation (17).


(17) $$F1=\left(\frac{2}{Recall^{-1}+Precision^{-1}}\right)=2\frac{Precision\cdot Recall}{Precision+Recall}$$

The Average Precision (AP) measures the mean highest precision at various recall levels per category. The mean Average Precision (mAP) averages these APs across categories, assessing object detection models’ overall performance. It is a comprehensive metric suitable for multi-category object detection tasks, as illustrated in the following formula, Equations (18) and (19).


(18) $$AP=\int_{0}^{1}P\left(r\right)dr$$

(19) $$mAP=\frac{\sum_{j=1}^{S}AP\left(j\right)}{S}$$

where S denotes the total category count, and the divisor is the aggregate of AP values across all categories.

Moreover, GFLOPs measures the computational complexity, allowing for efficiency comparisons among models. FPS, indicating images processed per second, assesses real-time performance. The formula involving $t_{avg}$ represents FPS calculation, as shown in Equation (20).


(20) $$FPS=\frac{1}{t_{avɡ}}$$

## 5 Results and analysis

### 5.1 Backbone network comparative experiment

The backbone network of the RT-DETR model, employing ResNet-18 for feature extraction, encompasses four Basic_Block modules. To explore the appropriate position for enhancing the backbone network architecture, the introduced PConv_Block module is utilized to substitute each Basic_Block module, and the performance of the enhanced model is examined.

As indicated in **Table 2**, the introduction of the PConv_Block module leads to a reduction in the computational cost of the model, while the highest mAP50 also increases by 1.3%. However, within the backbone network structure, it has been found that the number of PConv usages is not the more the better, after all 4 modules are improved mAP50 instead decreased by 0.5%, and experiments found that it is more effective to use it in the middle feature layer. This may be due to PConv requiring both location and semantic information, which are relatively balanced in the middle feature layers. Based on the experimental results, this paper replaces the last three Block modules in the backbone of the baseline model with PConv_Block modules, lightening the model structure while improving detection accuracy.

**Table 2:** Comparison of model performance after improving the backbone network.

Model	P(%)	R(%)	mAP50(%)	mAP50:95(%)	GFLOPs(G)
RT-DETR	81.7	78.7	82.9	69.8	57.3
RT-DERT+1 PConv_Block	84.2	77.9	82.5	69.3	53.8
RT-DERT+2 PConv_Block	84.8	78.5	84.1	70.5	50.2
RT-DERT+3 PConv_Block	85.1	80.9	84.2	71.3	46.7
RT-DERT+4 PConv_Block	82.9	77.6	82.4	68.8	43.2

Furthermore, the challenge in accurately detecting tomato ripeness involves minimizing computational demands while enhancing precision and speed. To corroborate the efficiency of employing partial convolution, several leading convolutional networks have been chosen for benchmarking in comparative studies, as illustrated in **Table 3**.

**Table 3:** Comparative experiment results of different backbone networks.

Backbone network	P(%)	R(%)	mAP50(%)	mAP50:95(%)	Params(M)	GFLOPs(G)
Basic_Block	81.7	78.7	82.9	69.8	19.97	57.3
DualConv_Block	84.3	80.1	83.6	69.5	16.02	50.2
AKConv_Block	82.2	77.6	81.5	67.5	15.44	49.4
DCNv2_Block	81.8	81.9	84.3	70.5	20.41	47.4
DySnakeConv_Block	83.8	80.6	84.0	68.2	27.96	61.2
PConv_Block	85.1	80.9	84.2	71.3	14.17	46.7

As can be seen from **Table 3**, compared with the original model, the introduction of lightweight PConv_ Block not only significantly reduces the model parameters and computational cost, but also achieves the improvement of P, R, and mAP, of which the mAP50 obtains a sizable improvement of 1.3 percentage points. The integration of Deformable Convolution v2 (Zhu et al., 2019) has resulted in an increase of 1.4% in mAP50, thus yielding enhanced accuracy; however, this enhancement comes at the cost of a 2.2% increase in the parameter count of the model. Additionally, the current study has incorporated DySnakeConv (Qi et al., 2023) into the original Basic_Block module. While DySnakeConv demonstrates impressive performance on the mAP50 metric, improving by 1.1% over the original model, it has substantially increased both the parameter count and computational demand of the model, with GFLOPs experiencing a 6.8% surge. In the final analysis, compared to the original model, the backbone integration of DualConv (Zhong et al., 2022) and AKConv (Zhang et al., 2023b), despite reducing both parameters and computational load, results in respective reductions of 0.3% and 2.3% in mAP50:95, which does not satisfy the high-precision requirements for tomato ripeness detection. Upon comparison of the performance of several prevalent operators, the PConv_Block proposed in this article exhibits exceptional performance.

### 5.2 Verifying the role of AIFI-DAT module

**Figure 7:**
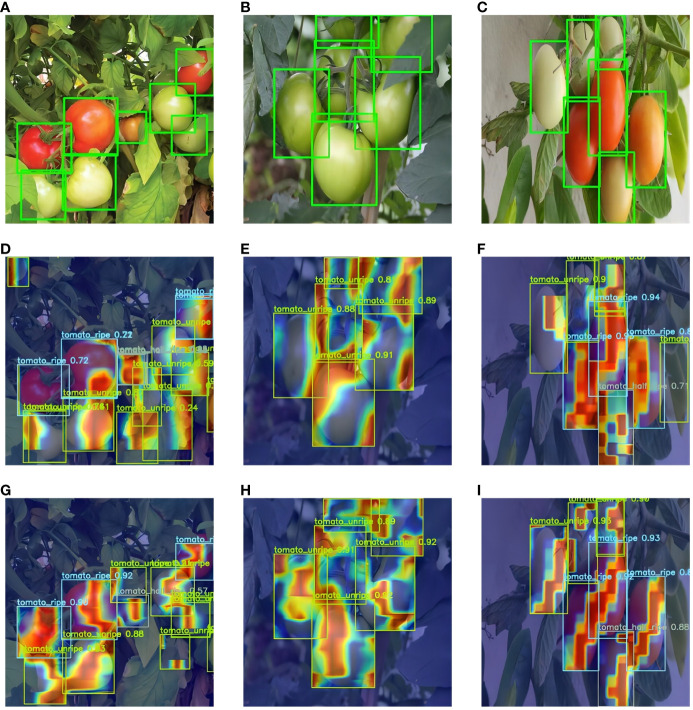
Comparison of feature visualization before and after adding the AIFI-DAT module. **(A–C)** image correctly labeled boxes, **(D–F)** feature heatmaps of the original RT-DETR, and **(G–I)** feature heatmaps with the addition of the AIFI-DAT module.

To confirm and explore the factors contributing to enhanced tomato ripeness detection performance through the integration of the intra-scale feature interaction module with deformable attention, this study utilized XGrad-CAM (Fu et al., 2020) for generating and contrasting heatmaps pre and post the incorporation of the attention mechanism. This comparative evaluation is depicted in **Figure 7**.

From the above figures, it can be observed that the RT-DETR model without the AIFI-DAT module neglects certain tomato targets, while attending to irrelevant locations such as tree leaves. Upon integration of the AIFI-DAT module, the focus of the model becomes more concentrated and accurate, mitigating attention towards interfering elements like foliage. Additionally, it enables precise identification of the ripeness of smaller tomatoes.

### 5.3 Verifying the effectiveness of the slimneck-SSFF structure

As seen in the ablation experiments in **Table 4**, by comparing four different configurations of the model, the specific effects of various neck network improvement strategies on the model performance can be observed. Model 2, which introduces slimneck, enhances the precision and recall by 1.2% and 2.5%, in comparison with the RT-DETR model, while the calculation complexity and the parameter count are diminished to 53.3 GFLOPs and 19.30 M, illustrating the significant effect of the slimneck strategy in boosting the computational efficiency of the model. Conversely, Model 3, employing the SSFF strategy, markedly improves the mAP50 and mAP50:95 by 2.1% and 3.1%, yet modestly elevates the computational complexity and the number of parameters, highlighting the additional resource requirement in aiming for higher detection accuracy. Ultimately, Model 4, merging both slimneck and SSFF strategies, attains equilibrium, raising the precision and recall to 85.4% and 82.4% while enhancing mAP50 and mAP50:95 by 2.1% and 2.6%, and trimming the number of parameters by 1.8%, thus achieving the optimization objective of managing the consumption of computational resources while preserving high performance.

**Table 4:** Performance comparison of models after neck improvements.

Model	P (%)	R (%)	mAP50 (%)	mAP50:95 (%)	GFLOPs (G)	Params (M)
1.RT-DETR	81.7	78.7	82.9	69.8	57.3	19.97
2.RT-DERT+slimneck	82.9	81.2	83.9	70.3	53.3	19.30
3.RT-DERT+SSFF	84.4	81.6	85.0	72.9	61.5	20.16
4.RT-DERT+slimneck+SSFF	85.4	82.4	85.0	72.4	57.6	19.61

### 5.4 Validity of loss function improvements

To substantiate the effectiveness of the proposed Inner-EIoU loss function, experiments were conducted by selecting different ratio values to alter the size of the auxiliary bounding box. Comparative analyses were performed against established loss functions such as GIoU, SIoU, EIoU, and Shape-IoU (Rezatofighi et al., 2019; Gevorgyan, 2022; Zhang et al., 2022; Zhang and Zhang, 2023). According to the data illustrated in **Table 5**, the model incorporating the Inner-EIoU loss function with a ratio of 0.7 manifested superior detection precision. Relative to the baseline model employing the GIoU loss function, there was an enhancement of 0.9% in the metric mAP50 and a 0.7% increment in mAP50:95. This suggests that the use of the Inner-EIoU loss function can lead to more stable regression on bounding boxes and higher prediction accuracy.

**Table 5:** Performance comparison of models with improved loss functions.

Loss function	P(%)	R(%)	mAP50(%)	mAP50:95(%)
GIoU	86.3	82.8	85.9	73.2
SIoU	85.0	83.5	85.5	72.8
Shape-IoU	86.7	82.4	85.5	73.0
EIoU	84.2	83.8	86.2	73.7
Inner-EIoU(ratio=0.70)	86.9	84.1	86.8	73.9
Inner-EIoU(ratio=0.75)	85.4	81.1	84.6	70.6
Inner-EIoU(ratio=0.80)	86.0	82.6	85.9	73.3
Inner-EIoU(ratio=1.10)	86.1	83.1	85.4	73.0
Inner-EIoU(ratio=1.13)	86.0	82.9	85.5	73.1
Inner-EIoU(ratio=1.15)	86.7	83.4	85.7	73.2

In models for detecting objects, the goal is to accurately determine the position and dimensions of objects with maximum precision. Therefore, a lower bounding box loss indicates a smaller discrepancy between the predicted bounding boxes and the true annotations, signifying enhanced model performance. From **Supplementary Figure 2**, it is evident that the Inner-EIoU markedly outperforms other loss functions, facilitating accelerated descent velocity and reducing the convergence time of the model.

### 5.5 Ablation study

To verify the enhancement effect of the proposed improvement modules on the model, eight sets of ablation experiments were designed. On the basis of the RE-DETR network, the following modifications were made: the Basic_Block in the feature extraction network was replaced with the lightweight PConv_Block module, the intra-scale feature interaction module equipped with a deformable attention mechanism was added, and the feature fusion network was optimized using slimneck and SSFF. Additionally, the loss function was switched to Inner-EIoU. The experiments were conducted by successively adding each improvement module, and the results are presented in **Table 6**.

**Table 6:** Results of ablation experiments.

Methods	PConv_ Block	AIFI- DAT	slimneck- SSFF	Inner- EIoU	mAP50 (%)	mAP50:95 (%)	GFLOPs (G)	Params (M)	FPS (f/s)
1.base	—	—	—	—	82.9	69.8	57.3	19.97	78.3
2	√	—	—	—	84.2	71.3	46.7	14.17	95.9
3	—	√	—	—	83.8	71.1	57.5	19.98	89.8
4	—	—	√	—	85.0	72.4	57.6	19.61	79.9
5	—	—	—	√	83.8	71.2	57.0	19.88	84.2
6	√	√	—	—	84.8	72.0	46.9	14.17	95.8
7	√	√	√	—	85.9	73.2	47.2	13.81	106.9
8.ours	√	√	√	√	86.8	73.9	47.2	13.81	108.6

Where “√” symbol represents the improvement of the structure of the ordinate corresponding to this symbol.

The ablation study results presented in **Table 6** elucidate that substituting the backbone with a module incorporating lightweight partial convolution engenders enhancements of 1.3%, 1.5%, and 22.5% across the mAP50, mAP50:95, and FPS metrics respectively. This evidences that refining the backbone network concurrently elevates accuracy and expedites detection velocity, while also effectuating a diminution in computational load and parameter count by 18.5% and 29.1%, respectively. The incorporation of the AIFI-DAT layer into the improved model, relative to its predecessor, yielded ascensions of 0.9% and 14.7% in mAP50 and FPS, respectively. Moreover, the integration of a deformable attention mechanism slightly augmented the computational and parameter requisites of the model, yet significantly bolstered its performance in discerning tomatoes of disparate shapes. The amalgamation of the slimneck-SSFF architecture within the neck network substantially uplifted mAP50 and mAP50:95 by 2.1% and 2.6%, correspondingly, while maintaining stability in other metrics. This attests to the efficacious enhancement of the fusion and articulation of tomato feature information by the slimneck-SSFF framework. Subsequent to adopting the Inner-EIoU loss function, the model manifested incremental enhancements of 0.9%, 1.4%, and 7.5% in mAP50, mAP95, and FPS, respectively.

Additionally, a comparison between Experiment 1 and Experiment 6 reveals that the model integrating the PConv_Block module and AIFI-DAT layer exhibited enhancements of 1.9%, 2.2%, and 22.3% in mAP50, mAP50:95, and FPS, respectively, alongside a substantial reduction in computational and parameter volume. Further analysis of Experiments 1, 6, and 7 shows that, following the incorporation of the slimneck-SSFF architecture in Experiment 7, there was a marginal increase in computational demand compared to Experiment 6, yet the precision and speed of the model were augmented. Finally, a comparison between Experiments 1 and 8 demonstrates that after implementing four model improvements, the proposed PDSI-RTDETR model, relative to the baseline model, achieved respective increases in mAP50, mAP50:95, and FPS metrics by 3.9%, 4.1%, and 38.7%, with a decrease of 17.6% in GFLOPs and a reduction of 6.16M in parameter count. A comprehensive analysis of **Table 6**, the results of the ablation study substantiate the efficacy of each proposed improvement module.

**Figure 8:**
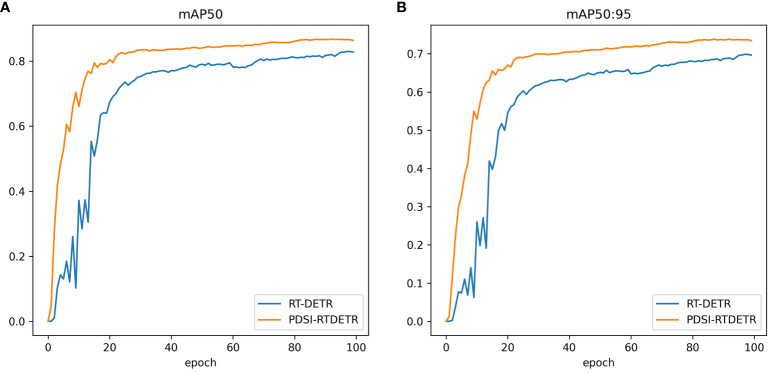
Comparison of mean Average Precision between RT-DETR and PDSI-RTDETR. **(A)** mAP50 comparison curve, **(B)** mAP50:95 comparison curve.

To more explicitly showcase the superiority of the proposed PDSI-RTDETR model, the training curves for mAP50 and mAP50:95 of both the original model and the improved model were visualized and compared, with blue representing the RT-DETR model and yellow denoting the PDSI-RTDETR model, as illustrated in **Figure 8**. It is evident from the graph that, across various epochs of training, the enhanced model consistently surpasses the original model in both mAP50 and mAP95 metrics, demonstrating a heightened precision and improved effectiveness in detecting the ripeness of tomatoes.

### 5.6 Comprehensive analysis of the improved model

**Table 7** delineates the performance of the enhanced model in detecting tomatoes at various stages of ripeness. Examining the overall performance, the model exhibits high precision and recall rates of 86.9% and 84.1%, respectively, achieving an mAP50 of 86.8% and an mAP50:95 of 73.9%, thereby affirming its effectiveness in adjudicating the ripeness of tomatoes comprehensively. Notably, in the detection of overripe and rotten tomatoes, the model demonstrates exceptionally high precision rates of 96.1% and 87.7%, respectively, along with superior scores in mAP50, reaching 93.4% and 89.7%, respectively. This signifies the robust capability of the model to recognize prominent feature alterations during the ripening process, such as intensified coloration and structural changes in the fruit. However, in identifying tomatoes at the half-ripe stage, the precision and recall rates of the model are marginally lower, with notably diminished mAP50 and mAP50:95 scores of 67.4% and 70.4%, respectively. This can be attributed to the subtler feature transitions characteristic of this ripening phase, presenting challenges to the detection capabilities of the model. Although the precision in detecting unripe tomatoes is the lowest at 80.6%, a higher recall rate of 90.1% suggests the efficiency of the model in not overlooking unripe tomatoes. Overall, the PDSI-RTDETR model demonstrates exemplary performance in tomato ripeness detection, particularly distinguishing itself in identifying overripe and rotten tomatoes, showcasing significant advantages.

**Table 7:** Performance of the improved model in detecting tomatoes of different ripeness levels.

Class	Instances	P(%)	R(%)	mAP50(%)	mAP50:95(%)
all	1925	86.9	84.1	86.8	73.9
tomato_half_ripe	216	85.3	67.4	70.4	60.0
tomato_overripe	328	96.1	92.8	93.4	81.5
tomato_ripe	627	85.0	84.1	88.7	72.9
tomato_rotten	265	87.7	86.1	89.7	81.0
tomato_unripe	489	80.6	90.1	91.6	74.1

**Figure 9:**
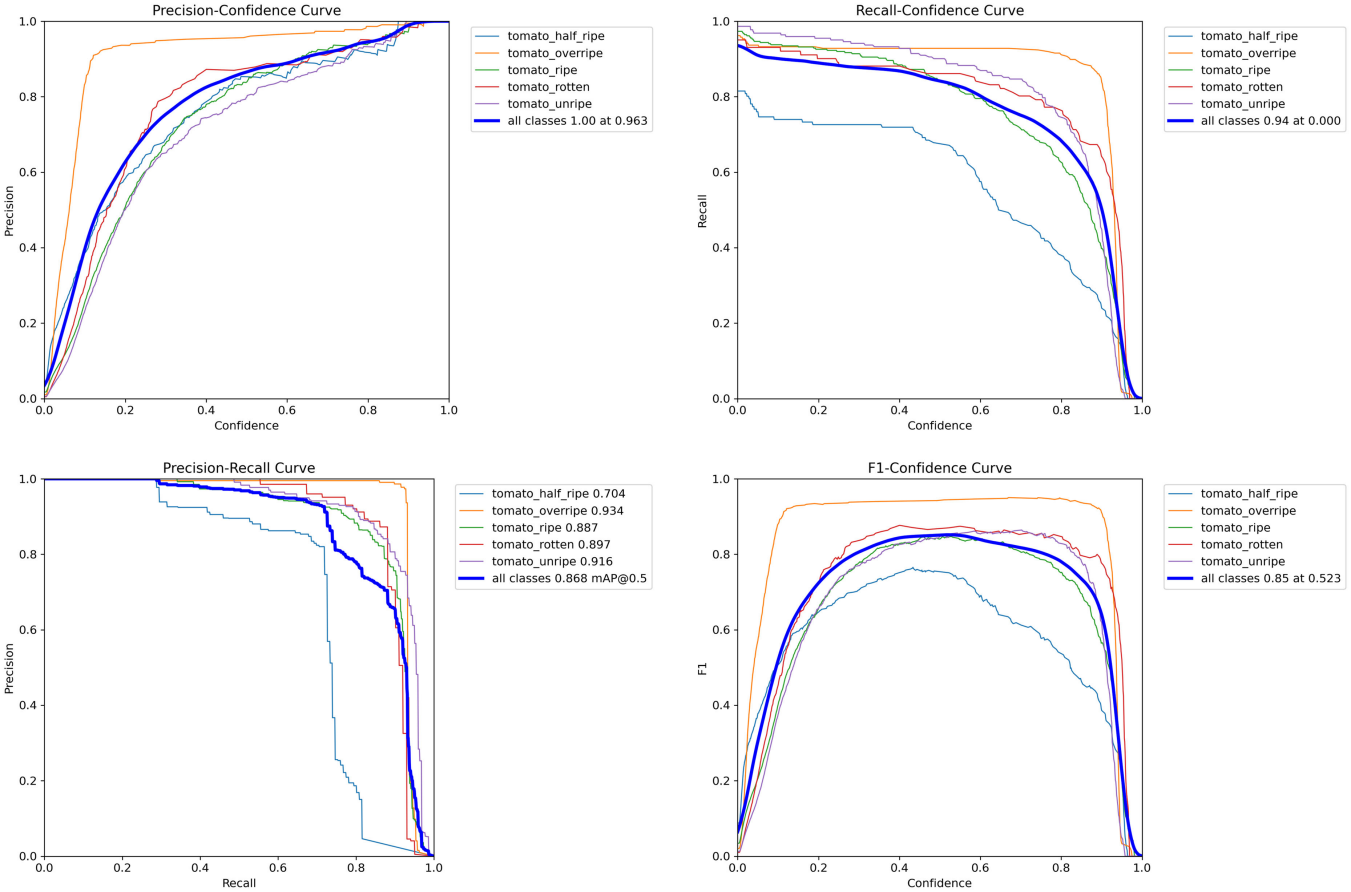
The precision, recall, mAP50, and F1 comparison curves for tomatoes at each ripeness level using the PDSI-RTDETR model.

To visually demonstrate the capability of the model in recognizing tomatoes at varying stages of ripeness, we visualized several evaluation metrics, as depicted in **Figure 9**. The comparison of performance curves for tomatoes of each ripeness category distinctly illustrates the detection abilities of the improved model across different maturation phases. Within **Figure 9** respectively represent the precision, recall, mAP50, and F1 comparison curves for tomatoes at each level of ripeness.

**Figure 10:**
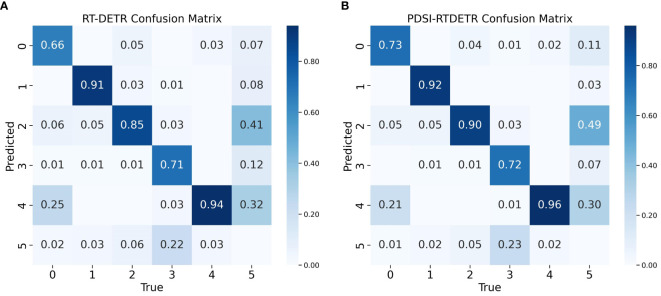
Comparison of model confusion matrix before and after improvement. **(A)** Confusion matrix for RT-DETR, **(B)** Confusion matrix for PDSI-RTDETR.

**Figure 10** presents the confusion matrices for the RT-DETR and PDSI-RTDETR models at their respective optimal performances. From the comparative visualization, it is intuitively observable that the PDSI-RTDETR model surpasses the original model in classification performance across all categories. Based on the analysis provided, the PDSI-RTDETR model proposed in this paper demonstrates exceptional performance in the task of tomato ripeness detection.

### 5.7 Comparison of different detection models

The performance of PDSI-RTDETR was compared with several other object detection models, including Faster-RCNN (Ren et al., 2016), SSD (Liu et al., 2016), YOLOv5, YOLOv6 (Li et al., 2022b), YOLOv8, and the RT-DETR series.

As indicated in **Table 8**, compared to Faster-RCNN, SSD, YOLOv5, YOLOv6, and YOLOv8, the mean average precision with IoU of 0.5 was found to be higher by 10.0%, 7.9%, 3.5%, 1.7%, and 1.4%, respectively. Furthermore, the detection time was recorded at 4.2 milliseconds per image, satisfying the criteria for real-time detection. Additionally, in comparison to the RT-DETR-L, RT-DETR-R34, and RT-DETR-R50 models, the precision of the proposed model was enhanced by 1.8%, 5.6%, and 3.8%, respectively. Compared to the original RT-DETR model, the mAP50 and mAP50:95 were increased by 3.9% and 4.1%, respectively, while F1 score increased by 5%. Consequently, in comparison to other object detection networks, PDSI-RTDETR demonstrated significant improvements in accuracy and speed for the detection of tomato ripeness within natural environments.

**Table 8:** Comparison of performance of different models.

Model	P(%)	R(%)	mAP50(%)	mAP50:95(%)	F1	Time(ms)
Faster R-CNN	76.8	75.7	76.8	62.5	0.76	4.1
SSD	79.2	75.8	78.9	64.5	0.77	5.9
YOLOv5	82.3	80.2	83.3	70.2	0.81	8.2
YOLOv6	85.8	81.8	85.1	69.6	0.83	5.6
YOLOv8	84.3	81.2	85.4	71.4	0.83	7.9
RT-DETR	81.7	78.7	82.9	69.8	0.80	8.5
RT-DETR-L	84.6	81.8	85.0	71.7	0.83	5.4
RT-DETR-R34	80.8	76.9	80.6	66.2	0.79	4.4
RT-DETR-R50	82.6	81.0	84.2	69.9	0.82	8.2
PDSI-RTDETR	86.4	84.1	86.8	73.9	0.85	4.2

The PDSI-RTDETR model introduced in this study outshines the baseline model in detecting tomato ripeness and surpasses several prevalent models for object detection, as shown in **Supplementary Figure 3**. An increase of 3.9% in mAP50 is noted when compared with the baseline RT-DETR model, alongside a decrease in image processing time by 4.2 milliseconds. These findings jointly suggest enhancements in both precision and processing efficiency by the model.

### 5.8 Visual analysis

**Figure 11:**
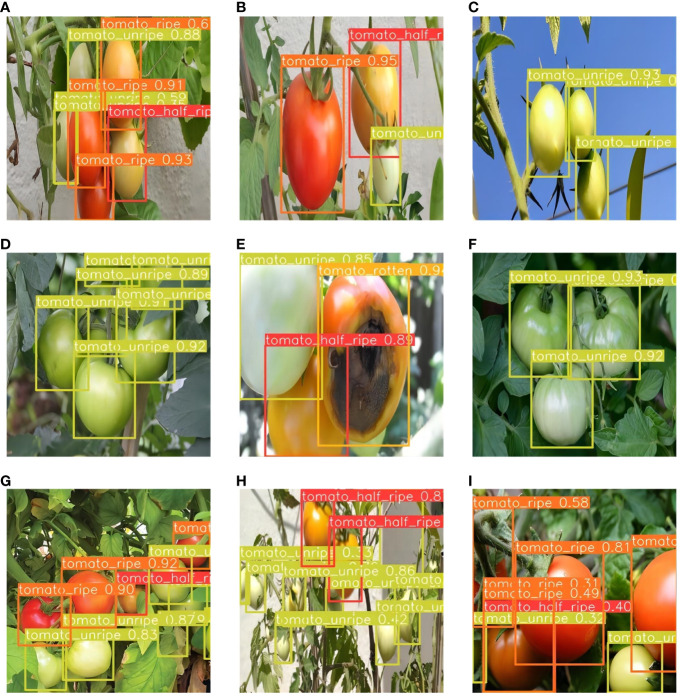
Examples of tomato ripeness detection results under different conditions. **(A–C)** under sunlight, **(D–F)** under shade, **(G–I)** under dense occlusion.

In natural settings, tomatoes are subjected to a variety of lighting conditions, including uneven illumination, potential obstructions by leaves or branches, and even instances of fruit overlap. To address these challenges, we evaluated the performance of our enhanced model across diverse scenarios. As illustrated in **Figure 11** , we present examples of tomato ripeness detection under various conditions. These findings demonstrate the exceptional robustness of the model in managing changes in lighting and in identifying overlapping fruits.

**Figure 12:**
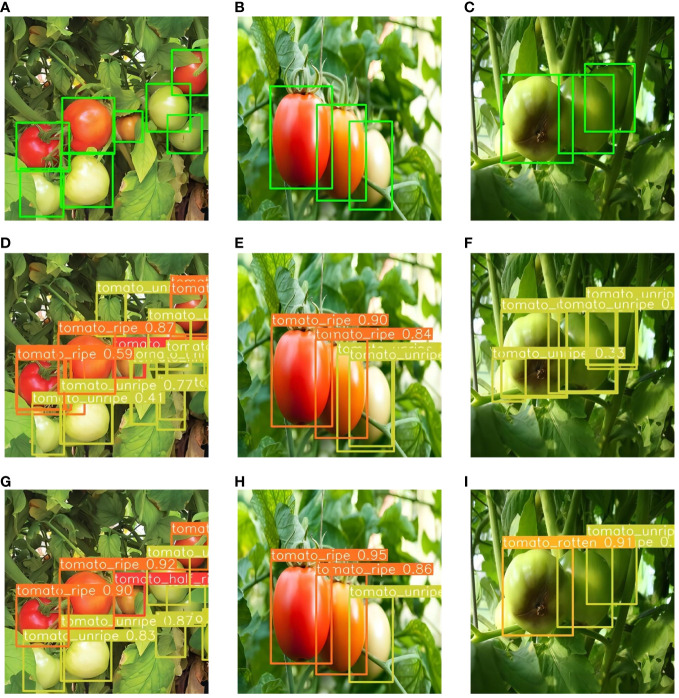
Partial detection results of two models. **(A–C)** Ground truth bounding boxes, **(D–F)** Predicted images from RT-DETR model, **(G–I)** Predicted images from PDSI-RTDETR.

**Figure 12** displays the comparative results of tomato ripeness prediction between the PDSI-RTDETR and the original RT-DETR models under varying environmental conditions.

Through these images, we can clearly observe that PDSI-RTDETR surpasses the original model in detection accuracy. Notably, a comparison presented in **Figure 12** vividly showcases the ability of the enhanced model to accurately distinguish the ripeness of tomatoes in dense areas. Moreover, **Figure 12D** reveal that the original model mistakenly identified some leaves as unripe tomatoes, and **Figure 12F** shows the misidentification of rotten tomatoes as unripe ones. In contrast, the improved PDSI-RTDETR model correctly detected the conditions, as shown in **Figures 12G**–**I**.

## 6 Discussion

The experimental results demonstrate that the proposed PDSI-RTDETR model achieves significant improvements in both accuracy and efficiency for tomato ripeness detection in natural environments. The ablation studies confirm the effectiveness of each proposed improvement module, including the lightweight PConv_Block, the AIFI-DAT module, the slimneck-SSFF structure, and the Inner-EIoU loss function.

The introduction of the PConv_Block module in the backbone network not only reduces the computational cost and parameter count but also improves the detection accuracy, which can be attributed to the ability of partial convolution to extract more discriminative features by focusing on informative regions while suppressing irrelevant background information. The AIFI-DAT module further enhances the feature representation by capturing multi-scale contextual information and adaptively adjusting the receptive field based on the object scale, as revealed by the visualization of attention maps using XGrad-CAM, which demonstrates that the AIFI-DAT module enables the model to focus more on the target tomatoes while suppressing the interference from background elements such as leaves. In the neck network, the slimneck-SSFF structure effectively balances the trade-off between accuracy and efficiency, with the slimneck strategy reducing the computational complexity and parameter count, while the SSFF strategy improves the feature fusion and representation capability, as demonstrated by the ablation experiments, which show that the combination of slimneck and SSFF achieves the best performance, indicating the complementary nature of these two strategies. Furthermore, the Inner-EIoU loss function promotes more precise bounding box regression by penalizing the mismatch between the predicted and ground-truth boxes, and the comparison with other state-of-the-art loss functions validates the superiority of Inner-EIoU in terms of convergence speed and detection accuracy.

A comprehensive analysis of the improved model reveals its strong capability in detecting tomatoes at different ripeness stages, particularly for unripe, overripe, and rotten tomatoes. The high precision and recall rates demonstrate that the model can capture the unique features associated with each ripeness stage. Comparisons with other object detection models, including Faster-RCNN, SSD, YOLOv5, YOLOv6, YOLOv8, and the RT-DETR series, highlight the superior performance of PDSI-RTDETR in terms of accuracy and speed. Visual analysis under various environmental conditions proves the model’s robustness in handling challenges such as uneven illumination, occlusion, and fruit overlap. When applied to intelligent harvesting robots in the future, this approach has the potential to enhance the quality of tomato harvesting by reducing the collection of immature and spoiled fruits.

## 7 Conclusion

This study proposes a fine-grained identification method for assessing tomato ripeness, which can provide more accurate decision support for tomato grading intelligent harvesting and management. In this paper, an efficient lightweight tomato ripeness detection model, PDSI-RTDETR, is proposed based on RT-DETR architecture. Substituting the original Basic_Block module of the backbone network with a streamlined PConv_Block module renders the model more compact, lowering computational requirements and the total parameter count. Then the deformable attention module is combined with AIFI module to enhance the ability of extracting detailed features of tomato and improve the accuracy and efficiency of the model. In addition, the slimneck-SSFF feature fusion architecture is proposed as the feature fusion network structure of the model, which utilizes the GSConv and VoVGSCSP modules to enhance the adaptability of the detector to tomatoes at different scales, generating more fine-grained semantic information while reducing the network computation. Ultimately, the original GIoU loss function is substituted by the Inner-EIoU loss function, which utilizes auxiliary frames to improve the ripeness determination accuracy for overlapping and small target tomatoes.

Extensive tests were performed to validate the efficacy of PDSI-RTDETR. Comparative results demonstrate that PDSI-RTDETR achieves enhancements in Precision, Recall, mAP50 and mAP50:95 by 4.7%, 5.4%, 3.9% and 4.1% respectively, compared to the RT-DETR. Concurrently, GFLOPs and parameters were decreased by 17.6% and 30.8% respectively, with the detection time per image being merely 4.2 milliseconds. Furthermore, the experiments confirmed the robustness of PDSI-RTDETR, evidencing its effective detection of tomato ripeness across various scenes involving different lighting and occlusion conditions. Future research will focus on integrating the model into a practical intelligent tomato harvesting robot for on-site validation. The harvesting system will be deployed in real tomato farming environments, and its performance will be evaluated under various field conditions. Furthermore, we plan to expand our research to explore ripeness detection methods for other fruits and agricultural products. By adapting the model architecture and training methodology, we aim to develop similar lightweight and efficient ripeness detection systems for a wider range of crops. Through these efforts, we strive to contribute to the development of intelligent and sustainable agricultural practices, improve the quality of harvested crops, reduce waste, and enhance agricultural efficiency.

## Data availability statement

The raw data supporting the conclusions of this article will be made available by the authors, without undue reservation.

## Author contributions

SW: Conceptualization, Methodology, Project administration, Software, Writing – original draft, Writing – review & editing. HJ: Funding acquisition, Resources, Writing – review & editing. JY: Conceptualization, Validation, Writing – original draft. XM: Formal analysis, Investigation, Writing – review & editing. JC: Data curation, Visualization, Writing – review & editing. ZL: Writing – original draft, Writing – review & editing. XT: Validation, Writing – review & editing.
