# Supplementary material for: Lightweight tomato ripeness detection algorithm based on the improved RT-DETR
Source: Front Plant Sci. 2024 Jul 5;15:1415297. doi: 10.3389/fpls.2024.1415297 (PMC11257922; doi:10.3389/fpls.2024.1415297)
Supplement: Supplementary file 1 [file DataSheet_1.docx]

Supplementary Material

Lightweight Tomato Maturity Detection Algorithm Based on the Improved RT-DETR

# Supplementary Figures and Tables

## Supplementary Figures


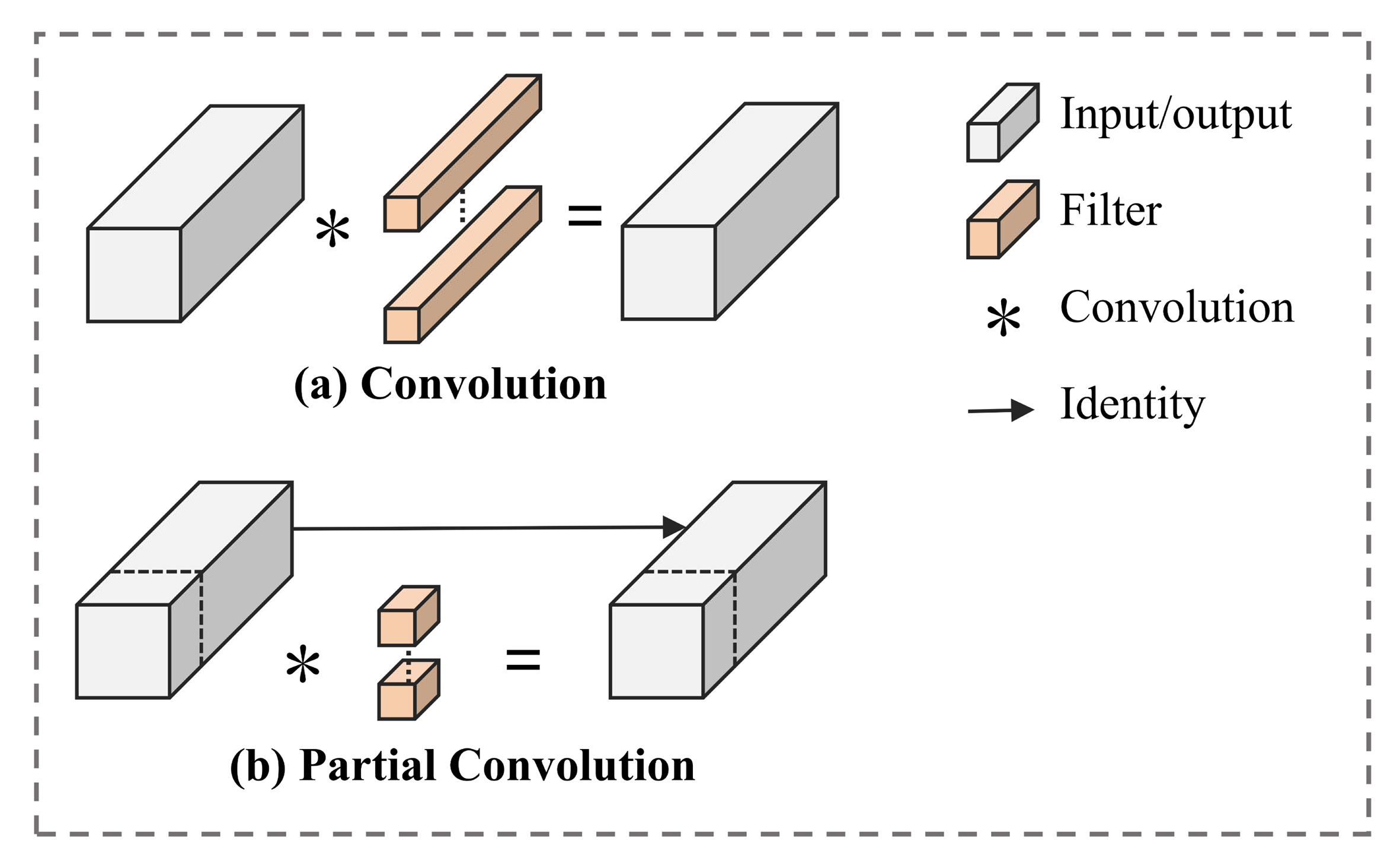


**Supplementary Figure 1.** Structural principle diagram of PConv.


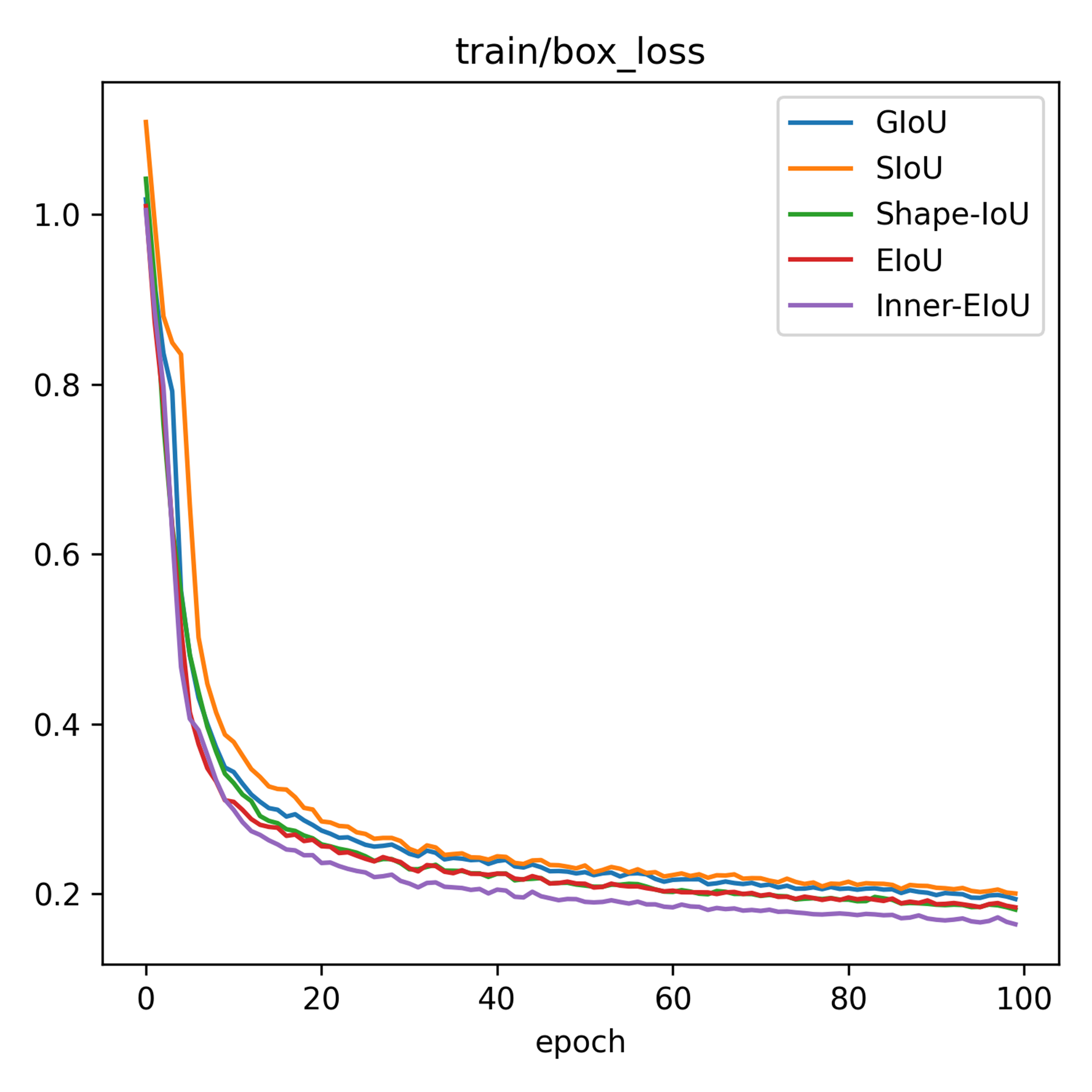


**Supplementary Figure 2.** Comparison curve of bounding box loss functions.


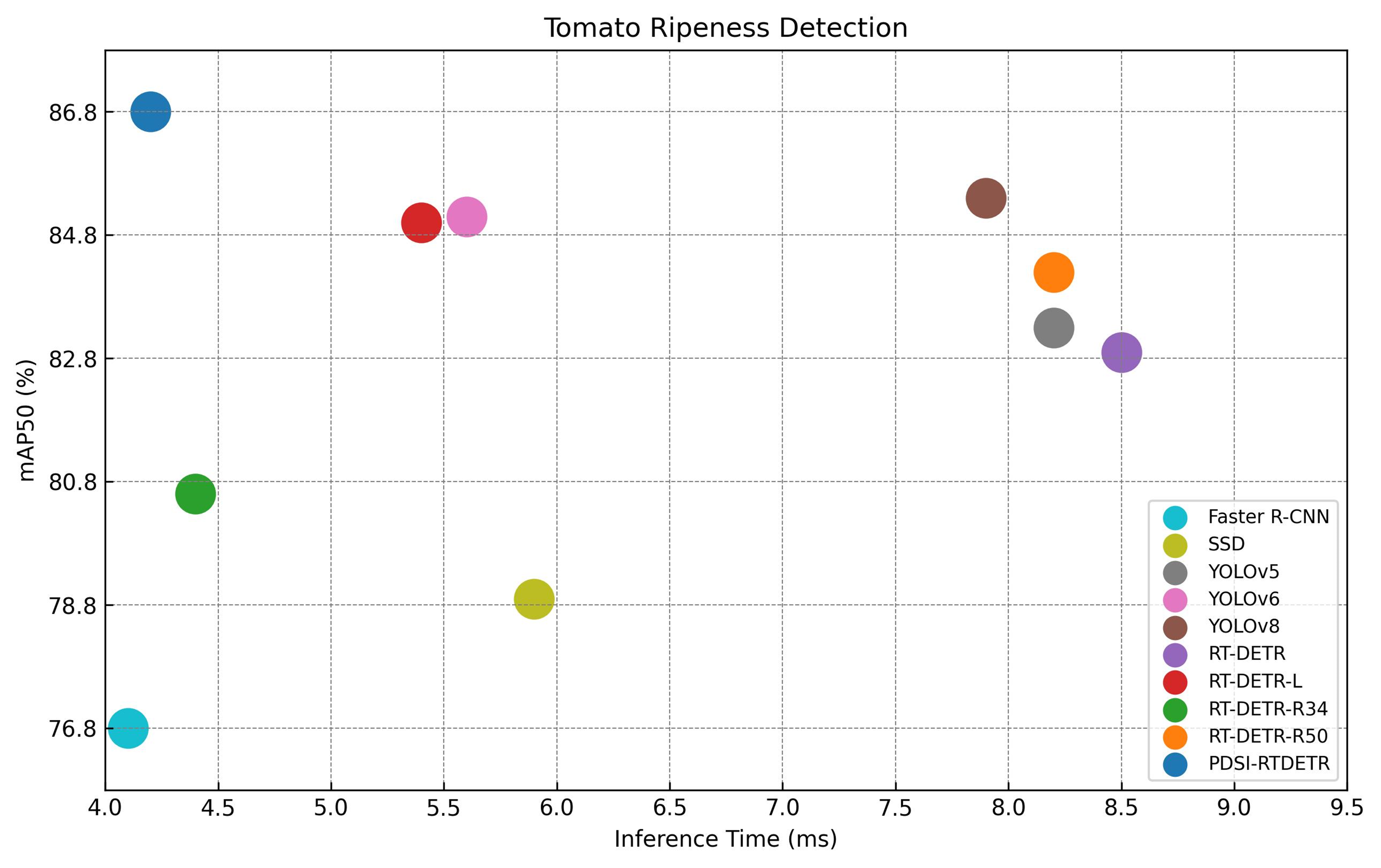


**Supplementary Figure 3.** Detection performance of different models (mean Average Precision vs. inference time).

## Supplementary Tables

**Supplementary Table 1.** Hardware configuration and model parameters.

| Types | Configuration | Types | Value |
| --- | --- | --- | --- |
| GPU | RTX 4090 | learning rate | 1E-4 |
| CPU | Intel 4310 | momentum | 0.9 |
| CUDA | 11.7 | optimizer | AdamW |
| CuDNN | 10.2 | batch | 4 |
